# Graph Convolutional Network and Convolutional Neural Network Based Method for Predicting lncRNA-Disease Associations

**DOI:** 10.3390/cells8091012

**Published:** 2019-08-30

**Authors:** Ping Xuan, Shuxiang Pan, Tiangang Zhang, Yong Liu, Hao Sun

**Affiliations:** 1 School of Computer Science and Technology, Heilongjiang University, Harbin 150080, China; 2 School of Mathematical Science, Heilongjiang University, Harbin 150080, China

**Keywords:** graph convolutional network, convolutional neural network, lncRNA-disease association prediction, attention mechanism at node feature level

## Abstract

Aberrant expressions of long non-coding RNAs (lncRNAs) are often associated with diseases and identification of disease-related lncRNAs is helpful for elucidating complex pathogenesis. Recent methods for predicting associations between lncRNAs and diseases integrate their pertinent heterogeneous data. However, they failed to deeply integrate topological information of heterogeneous network comprising lncRNAs, diseases, and miRNAs. We proposed a novel method based on the graph convolutional network and convolutional neural network, referred to as GCNLDA, to infer disease-related lncRNA candidates. The heterogeneous network containing the lncRNA, disease, and miRNA nodes, is constructed firstly. The embedding matrix of a lncRNA-disease node pair was constructed according to various biological premises about lncRNAs, diseases, and miRNAs. A new framework based on a graph convolutional network and a convolutional neural network was developed to learn network and local representations of the lncRNA-disease pair. On the left side of the framework, the autoencoder based on graph convolution deeply integrated topological information within the heterogeneous lncRNA-disease-miRNA network. Moreover, as different node features have discriminative contributions to the association prediction, an attention mechanism at node feature level is constructed. The left side learnt the network representation of the lncRNA-disease pair. The convolutional neural networks on the right side of the framework learnt the local representation of the lncRNA-disease pair by focusing on the similarities, associations, and interactions that are only related to the pair. Compared to several state-of-the-art prediction methods, GCNLDA had superior performance. Case studies on stomach cancer, osteosarcoma, and lung cancer confirmed that GCNLDA effectively discovers the potential lncRNA-disease associations.

## 1. Introduction

Long non-coding RNAs (lncRNAs) are non-coding RNAs with more than 200nt (nucleotides) in length [1]. There is mounting evidence that lncRNAs participate in the development and progression of numerous diseases [2,3]. Mutations and disorders of lncRNAs are associated with breast and colon cancer, atherosclerosis, and neurodegenerative diseases [4,5,6,7]. Therefore, identification of disease-related lncRNAs may help elucidate pathogenesis.

Computational biology techniques are essential and often used in many fields of biomedicine, ranging from the discovery of biomarkers to the development of drugs [8]. Machine learning and deep learning are being increasingly used to solve the most challenging problems [9,10,11,12,13,14,15]. In recent years, computational methods have been proposed to predict the associations between diseases and lncRNAs. These techniques can reliably screen disease-related lncRNA candidates. One forecasting method is the use of biological information related to the lncRNAs to infer potential lncRNA-disease associations such as genome location and tissue specificity. The lncRNAs near each other in the genome are often associated with similar diseases. Thus, Chen et al. and Li et al. proposed methods for predicting lncRNA-disease associations using genomic location data [16,17]. However, they cannot be applied to lncRNAs without first identifying the adjacent genes. Liu et al. and Biswas et al. used tissue specificity to predict potential disease-related lncRNAs [18,19]. However, this approach does not work for diseases without related tissue-specific gene records and cannot, therefore, predict their potential related lncRNAs.

Another forecasting method is based on machine learning prediction. Chen et al. developed a computational model based on Laplacian regularized least squares (LRLSLDA) to predict lncRNA-disease associations [20]. Chen et al. and Huang et al. optimized the similarity calculation method based on LRSLDA to improve its prediction performance [21,22,23]. However, these methods did not integrate multiple biological data related to the lncRNAs. The bipartite network was constructed using known lncRNA-disease associations to predict the potential lncRNA-disease associations [24,25]. Nevertheless, these methods are ineffective for diseases without known related lncRNAs. Potential lncRNA-disease associations are also inferred from random walk algorithms in heterogeneous networks containing disease and lncRNA nodes [26,27,28,29,30]. On the other hand, these methods depend on network topology data and the prediction results are biased towards disease nodes known to be associated with several lncRNAs.

Forecasting may also be performed by integrating various data sources related to lncRNAs or diseases such as the proteins and micro RNAs (miRNAs) interacting with lncRNAs and proteins associated with disease and so on. Lan et al. used the Karcher mean to merge numerous lncRNA and disease similarities calculated from multiple data sources [31,32]. They then identified potential lncRNA-disease associations based on a bagging support-vector machine (SVM) [32]. Certain matrix factorization-based prediction methods merge various data related to lncRNA, disease, and proteins [33,34]. However, none of the forecasting methods mentioned in this paragraph deeply integrate the topology information of the heterogeneous network.

In this study, we propose a model based on the graph convolution and convolution neural network, named GCNLDA, to predict potential lncRNA-disease associations. GCNLDA makes full use of topological information of lncRNA-disease-miRNA heterogeneous networks and data of similarities, correlations, and interactions among lncRNAs, diseases, and miRNAs. We constructed a heterogeneous network composed of lncRNA, miRNA, and disease nodes. The nodes were connected based on their similarities, associations, and interactions. We also constructed an embedding matrix of lncRNA-disease node pairs based on several biological premises regarding the probable associations between lncRNAs and diseases. A new framework based on a graph convolution and convolution neural network was developed to learn the network—and local representations of lncRNA-disease node pairs. The frame was made of two parts—the left and the right. On the left side of the framework, the autoencoder based on the graph convolution combines the attention mechanism of the node feature level to integrate the topological information of the heterogeneous lncRNA-disease-miRNA network. The right side of the framework focuses on learning the local representation of the lncRNA-disease node via the correlations among similarity, association, and interaction. A fivefold cross-validation showed that GCNLDA performance is significantly superior to other state-of-the-art prediction methods. Case studies on stomach cancer, osteosarcoma, and lung cancer confirmed that GCNLDA may successfully infer potential disease-associated lncRNA candidates.

## 2. Materials and Methods

### 2.1. Dataset for lncRNA-Disease Association Prediction

Data of lncRNA disease associations, lncRNA-miRNA interactions, and miRNA-disease correlations were obtained from previous reports [33]. Fu et al. extracted data for 2687 lncRNA-disease associations from LncRNADisease, lnc2cancer, and GeneRIF databases [16,35,36]. The original 1002 lncRNA-miRNA interaction and 5218 miRNA-disease association data were obtained from Starbase and the Human microRNA Disease Database (HMDD), respectively [37,38]. Semantic disease similarities were derived from the Dincrna database [39]. The associations, interrelationships, and similarities were compiled for 240 lncRNAs, 402 diseases, and 495 miRNAs.

### 2.2. Prediction Method Based on Graph Convolutional Network and Convolutional Neural Network

Our goal was to predict potential lncRNA-disease associations. A heterogeneous node network including lncRNA, disease, and miRNA was constructed. The embedding matrix of the lncRNA-disease node pairs was constructed based on several biological premises. The graph convolutional network module combined with the attention mechanism on the left side of the framework learned the network representation of the lncRNA-disease node pair. The convolutional neural network on the right side of the framework learned the local representation of the lncRNA-disease node pair. A combined strategy was used to obtain the final likelihood score of the association between the lncRNA and the disease. Here, the process is described using the lncRNA $l_{2}$ and the disease $d_{4}$ as examples.

#### 2.2.1. Construction of the lncRNA-Disease-miRNA Network 

A heterogeneous network was constructed and named LncDisMirNet. It consisted of the nodes lncRNA, miRNA, and disease. The LncDisMirNet comprised the lncRNA network (LncNet), the disease network (DisNet), the miRNA network (MirNet), and three types of connecting edges; which respectively represent the interaction between lncRNAs and miRNAs, the association between lncRNAs and diseases, and the association between miRNAs and diseases.

#### 2.2.2. Construction of the lncRNA, miRNA, and Disease Networks 

**Figure 1:**
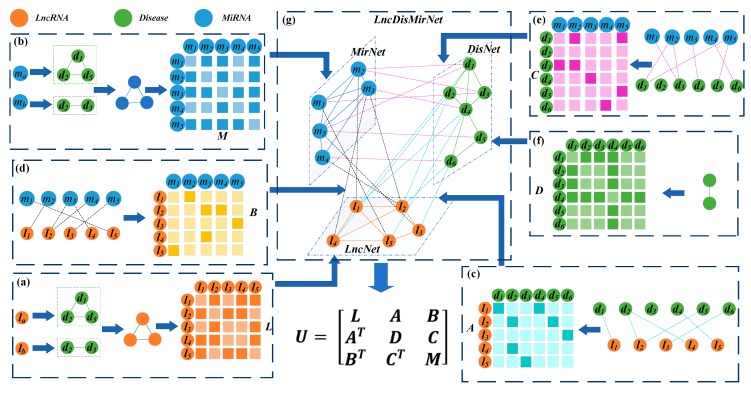
Construction and representation of a heterogeneous network with three different nodes. (**a**) LncRNA network (LncNet) and its adjacency matrix ***L*** were constructed by calculating the functional similarity of the lncRNAs according to their associated diseases. (**b**) Calculation of the functional similarity of the lncRNAs based on their related diseases and construction of miRNA network (MirNet) and the adjacency matrix ***M***. (**c**) Establishment of the connexion between LncNet and disease network (DisNet) based on known lncRNA-disease associations and construction of the adjacency matrix ***A***. (**d**) Connexion of LncNet and MirNet according to known interactions between lncRNAs and miRNAs and construction of the adjacency matrix ***B***. (**e**) Connexion of the miRNAs and diseases according to known miRNA-disease associations and construction of the adjacency matrix **C**. (**f**) Computation of the similarities based on the DAGs of the diseases and construction of DisNet and the adjacency matrix ***D***. (**g**) LncNet, DisNet, MirNet, and the connexions among them were used to construct the heterogeneous network LncDisMirNet and its adjacency matrix ***U***.

Two lncRNAs are usually associated with similar diseases if their functions are similar. Chen et al. calculated the functional similarity among lncRNAs [21]. To construct the lncRNA network, the similarity between two lncRNA nodes was determined by Chen’s method and an edge was added to connect them when their similarity was > 0. The weight of the edge was set to the similarity value (Figure 1a). The matrix $L=\left[L_{ij}\right]\;\in \;R^{N_{l}\times N_{l}}$ denotes LncNet, where $L_{ij}$ is the similarity between $l_{i}$ and $l_{j}$ and $N_{l}$ is the number of lncRNAs.

The same method was applied to determine the similarity between miRNAs and construct the network MirNet composed of miRNA nodes (Figure 1b). The matrix $M=\left[M_{ij}\right]\;\in \;R^{N_{m}\times N_{m}}$ was used to represent the MirNet with $N_{m}$ miRNA nodes. $M_{ij}$ represents the similarity between miRNA $m_{i}$ and $m_{j}$.

Wang et al. calculated the similarity between two diseases [40]. This method represented a disease by using a directed acyclic graph (DAG) comprising all annotations related to it. Here, disease similarity was used to construct the DisNet network, and the matrix $D\;=\left[D_{ij}\right]\;\in \;R^{N_{d}\times N_{d}}$ represented it. $D_{ij}$ represents the similarity between disease $d_{i}$ and disease $d_{j}$, and $N_{d}$ is the number of diseases (Figure 1f).

The connexion between the LncNet and DisNet nodes was established using the known lncRNA-disease correlation data. If the lncRNA node in LncNet is associated with a disease node in DisNet, an edge is added to connect them. The matrix $A=\left[A_{ij}\right]\in \;R^{N_{l}\times N_{d}}$ denotes the set of edges. When $A_{ij}=1$, there is an association between lncRNA $l_{i}$ and disease $d_{j}$. When $A_{ij}=0$, there is no association between them (Figure 1c).

Connexions between LncNet and MirNet and between DisNet and MirNet were established based on the data of the lncRNA-miRNA interaction and the miRNA-disease association. If lncRNA $l_{i}$ (disease $d_{i}$) in LncNet (DisNet) interacts (associate) with miRNA $m_{l}$ in MirNet, then $B_{ij}(C_{ij})=1$. If not, then $B_{ij}(C_{ij})=0$. The matrices $B=\left[B_{ij}\right]\in \;R^{N_{l}\times N_{m}}$ and $C=\left[C_{ij}\right]\in \;R^{N_{d}\times N_{m}}$ represented the connexions between LncNet and MirNet and between DisNet and MirNet, respectively (Figure 1d,e).

The heterogeneous network LncDisMirNet was constructed by combining LncNet, DisNet, and MirNet. LncDisMirNet is denoted by the matrix $U=\left[U_{ij}\right]\in \;R^{N\times N}$,

(1) $$U=\;\left[\begin{array}{ccc} L & A & B\\ A^{T} & D & C\\ B^{T} & C^{T} & M \end{array}\right],$$

where $N=\;N_{l}+\;N_{d}+N_{m}$, and $A^{T}$, $B^{T}$, $C^{T}$ are transpose matrices of ***A***, ***B***, and ***C***, respectively (Figure 1g).

#### 2.2.3. Attention Mechanism on the Left Side of the Framework 

The attention mechanism in a deep learning technique is similar to the visual attention mechanism in humans. The core goal was to select the information that was more critical to a given task. By applying our proposed attention mechanism, each feature of the nodes is assigned a different weight.

**Figure 2:**
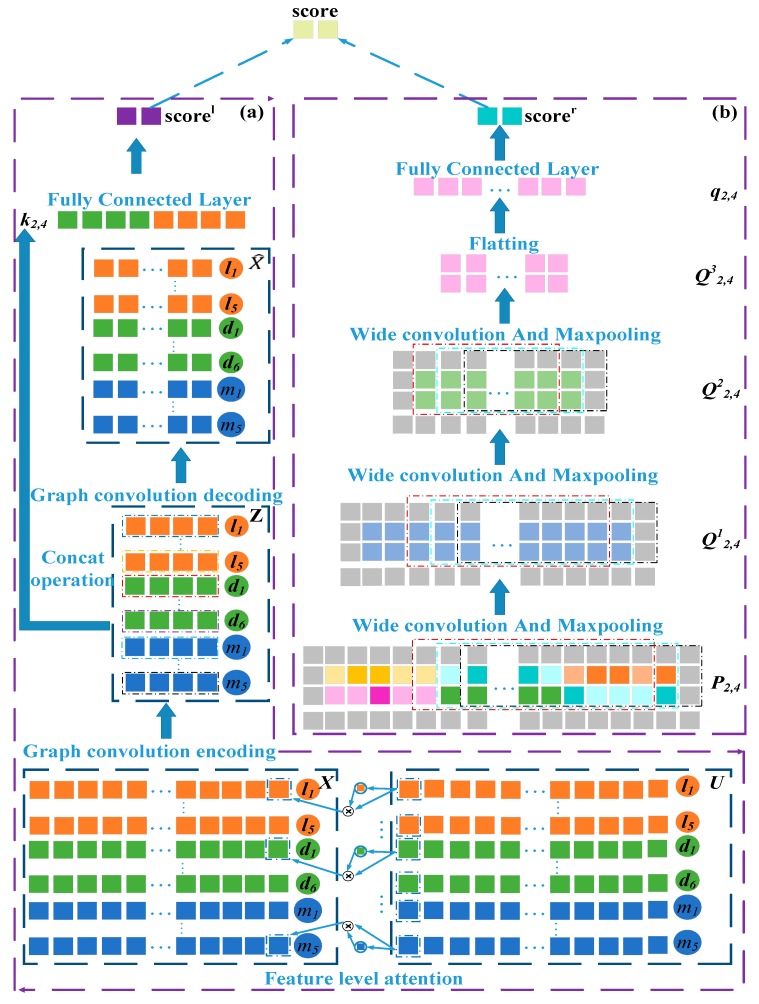
Overall model structure. (**a**) Establish the attention mechanism at the feature levels and the autoencoder based on graph convolution. (**b**) Construct the convolutional and pooling layers.

As shown in Figure 1g, the *i*^th^ row $u_{i}=\left(u_{i1},u_{i2},u_{i3},\ldots ,u_{iN}\right)$ in U reflects the topology information between the *i*^th^ node and all others in the network. For example, $u_{2}$ contains similarity links between lncRNA $l_{2}$ and $l_{1}\ldots l_{5}$, association links between $l_{2}$ and disease $d_{1}\ldots d_{6}$, and interaction links between $l_{2}$ and miRNA $m_{1}\ldots m_{5}$. Similarly, $u_{9}$ contains the links of disease $d_{4}$ to all lncRNAs, diseases, and miRNAs. Therefore, $u_{i}$ is the topology feature vector of the *i*^th^ node in LncMirDisNet. The topology feature vector of the $l_{2}$ node is $u_{2}$ and that for the $d_{4}$ node is $u_{9}$ (Figure 2).

The various features of the lncRNA and disease nodes contribute differently and uniquely to the association prediction. Thus, an attention mechanism was established at the node feature level to extract the important features of the $l_{2}-d_{4}$ association prediction. The attention scores of each node feature are defined as follows,

(2) $$s_{i}=\;H^{att}f\left(W^{att}u_{i}+\;b^{att}\right),$$

where $H^{att}\in \;R^{N\times N}$ and $W^{att}\in \;R^{N\times N}$ are parametric matrices, $b^{att}\in \;R^{N}$ is a bias vector and $f\left(t\right)=\tanh\left(t\right)=\;\frac{e^{t}-e^{-t}}{e^{t}+e^{-t}}$ is the activation function. The vector $s_{i}=\left[s_{i,1},s_{i,2},\ldots ,s_{i,j},\ldots ,s_{i,N}\right]$ is the attention score vector of each feature of $u_{i}$, where $s_{i,j}$ is the attention score of the *j*^th^ feature of $u_{i}$. $Softmax{\left(t\right)}_{k}=\;\frac{e^{t_{k}}}{\sum_{j}e^{t_{j}}}$ was used to normalize the attention scores for all features of $u_{i}$,

(3) $$\alpha_{i,k}=\;\frac{\exp\left(s_{i,k}\right)}{\sum_{j}\exp\left(s_{i,j}\right)},$$

where $\alpha_{i}=\left[\alpha_{i,1},\alpha_{i,2},\ldots ,\alpha_{i,k},\ldots ,\alpha_{i,N}\right]$ is the feature-level attention weight vector of $u_{i}$, and $\alpha_{i,k}$ is the weight of the *k*^th^ feature of $u_{i}$. Therefore, the node enhancement vector based on the feature-level attention mechanism is,

(4) $$x_{i}=\;\alpha_{i}\;ⓧ\;u_{i},$$

where ⓧ is the element-wise product operator and $x_{i}$ is the enhancement vector of $\;u_{i}$. The enhancement vectors of the lncRNA node $l_{2}$ and the disease node $d_{4}$ are $x_{2}=\;\alpha_{2}\;ⓧ\;u_{2}$ and $x_{9}=\;\alpha_{9}\;ⓧ\;u_{9}$, respectively.

#### 2.2.4. Graph Convolutional Network Module on the Right Side of the Framework

The graph convolutional network is a multilayer neural network proposed by Tomas Kpif in 2017 [41]. It uses the graph as an input, integrates the neighborhood node feature and structure information of the graph nodes, and represents them as a vector. Graph convolutional networks have been successfully applied towards the prediction of multidrug side effects, social networks, recommendation system and prediction of drug-target interactions [42,43,44,45]. Here, the graph convolutional network was used to predict lncRNA-disease associations. The heterogeneous network LncDisMirNet has connexions based on lncRNA, disease, and miRNA similarity, lncRNA-disease and miRNA-disease associations, and lncRNA-miRNA interactions. These are consistent so the entire heterogeneous network ***U*** is used as the input for the graph convolution.

First, $\hat{U}=\;U+I$ is the adjacency matrix with added self-connections, where I is the identity matrix. Then a symmetric Laplace normalization was performed on $\hat{U}$ to get $\tilde{U}\in \;R^{N\times N}$,

(5) $$\tilde{U}=\;E^{-\frac{1}{2}}\;\hat{U}\;E^{-\frac{1}{2}},$$

where $E\in \;R^{N\times N}$ is a diagonal matrix such that $E_{ii}=\;\sum_{j}{\hat{U}}_{ij}$, E is actually the degree matrix of $\hat{U}$. The graph convolution autoencoder takes in the structure matrix $\tilde{U}$ and the node feature matrix X as inputs. And the graph convolution autoencoder encodes the nodes in LncDisMirNet to obtain network representations of the lncRNA, disease, and miRNA nodes,

(6) $$Z=f\;\left(X,\tilde{U}\right)=Softmax\left(\tilde{U}\;X\;W^{enco}\right),$$

where $W^{enco}\;\in \;R^{N\times n}$ is a weight matrix and n is a hyper-parameter. The matrix $\tilde{U}$ is multiplied by X. This operation can be understood as an aggregation of spatial information. If K = $\tilde{U}X$, where $K_{i}\in R^{N}$, the *i*^th^ row in the matrix $K\in R^{N\times N}$ can be understood as the feature vector of the *i*^th^ node. K and $W^{enco}$ are multiplied to map the nodes to the low-dimensional vector $z_{i}\in \;R^{n}$. As shown in Figure 2, the second row $z_{2}$ and the ninth row $z_{9}$ in the matrix are network representations of $l_{2}$ and $d_{4}$, respectively.

Furthermore, we traced $z_{i}$ back to its original feature space. Z was subsequently decoded on the basis of the graph convolution,

(7) $$\hat{X}=\hat{f}\;\left(Z,\tilde{U}\right)=Sigmoid\left(\tilde{U}\;Z\;W^{deco}\right).$$

$W^{deco}\in \;R^{n\times N}$ is a parameter matrix and $Sigmoid\left(t\right)=\;\frac{1}{1\;+\;e^{t}}$ is the activation function. To make $\hat{X}$ and X as consistent as possible, the loss function of the graph convolution autoencoder was defined as MSE (mean-square error),

(8) $$L=\;\;\frac{\sum_{i}\sum_{j}{\left(X\left(i,j\right)-\;\hat{X}\left(i,j\right)\right)}^{2}}{N*N}.$$

The network representations $z_{i}$ of the lncRNA nodes and $z_{j}$ of the disease nodes obtained by graph convolutional neural networks were then combined to obtain the network representation $k_{i,j}\in \;R^{2*n}$ of the node pairs $l_{i}$-$d_{j}$,

(9) $$k_{i,j}=\;z_{i}\;⊕\;z_{j}.$$

As shown in Figure 2, the second row $z_{2}$ and the ninth row $z_{9}$ in the matrix are network representations of $l_{2}$ and $d_{4}$, respectively. $z_{2}$ and $z_{9}$ were concatenated to get $k_{2,9}$ and then projected onto a C (C = 2) class association probability distribution using fully connected and softmax layers. In this two-class distribution ***p^l^***, class 0 means that $l_{2}$ and $d_{4}$ are not associated whilst class 1 indicates association between $l_{2}$ and $d_{4}$. The probability of class 1 was taken as the predictive $score_{2,4}^{l}$ of the association between $l_{2}$ and $d_{4}$,

(10) $$score_{2,4}^{l}=\mathrm{softmax}\;\left(\;W^{l}\;k_{2,9}+b^{l}\right),$$

where $W^{l}\in \;R^{2\times \left(2*n\right)}$ is the parameter matrix of the fully connected layer and $b^{l}\in \;R^{2}$ is the bias term. $score_{2,4}^{l}$ measures the likelihood of association between lncRNA $l_{2}$ and disease $d_{4}$, and the greater its value, the more likely they are to be associated. The probability $score_{i,j}^{l}$ in which $l_{i}$ and $d_{j}$ may be correlated can be obtained by the same method.

#### 2.2.5. Construction of the Embedding Matrix of lncRNA-Disease Node Pairs 

**Figure 3:**
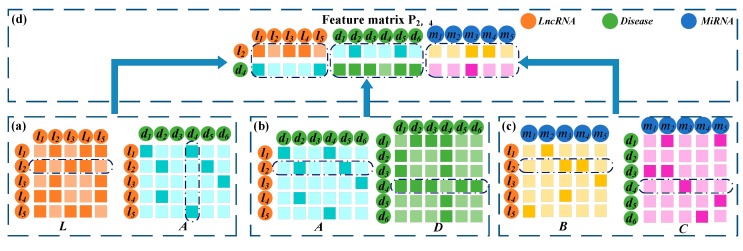
Construction of the embedding matrix of $l_{2}$-$d_{4}$ pair. (**a**) Construction of the first part of the embedding matrix based on the similarity between $l_{2}$ and the other lncRNAs and the association between $d_{4}$ and all lncRNAs. (**b**) The second part of the embedding matrix was constructed based on the similarity between $l_{2}$ and the other lncRNA and the association between $d_{4}$ and the other diseases. (**c**) Construction of the third part using the lncRNA-miRNA interactions and miRNA-disease associations. (**d**) Construction of the final embedding matrix $P_{2,4}$ by combining the representations of the first, second, and third parts.

The $l_{2}$ and $d_{4}$ serve to illustrate the process of constructing embedding matrix as shown in Figure 3. If $l_{2}$ and $d_{4}$ have similarities and associations with common lncRNAs, the likelihood of association between them is high. In the matrices ***L*** and ***A***, $l_{2}$ and $d_{4}$ have similarities and associations, respectively, with $l_{1}$. Thus, there may be an association between them. The second row of ***L*** records the similarity between $l_{2}$ and all lncRNAs. The fourth column of **A** records the associations between $d_{4}$ and all lncRNAs. These were spliced together as the first part of the embedding matrix $P_{2,4}\in \;R^{2\times N}$. Similarly, if $l_{2}$ and $d_{4}$ have connexions with common miRNAs and diseases, they are more likely to be associated. The second row of ***A*** and the fourth row of ***D*** were combined as the second part of $P_{2,4}$. Finally, the second row of ***B*** and the fourth row of ***C*** were combined as the third part of $P_{2,4}$. So far, lncRNA similarity, disease similarity, lncRNA-disease association, lncRNA-miRNA interaction, and disease-miRNA association were integrated to construct the embedding matrix $P_{2,4}$ of the node pair $l_{2}$-$d_{4}$. The same method is used to construct the embedding matrix $P_{i,j}$ for the other lncRNA-disease node pairs $l_{i}$-$d_{j}$.

#### 2.2.6. Convolutional Neural Networks Module on the Left Side of the Framework 

The embedding matrix $P_{i,j}$ of node pairs $l_{i}$-$d_{j}$ served as the input of the convolutional neural network to learn the local representation of $l_{i}$-$d_{j}$. To learn the marginal information of $P_{i,j}$ during the convolution process, a zero-padding operation was run on $P_{2,4}$ to obtain $P_{2,4}^{\text{′}}\in \;R^{T\times N_{1}}$, to be precise, pad zeros around $P_{2,4}$ were operated, where T=2+2 and $N_{1}=N+2$. In the first convolution layer, the filter length and width were set to $n_{f}$ and $n_{d}$, respectively. If the number of filters is $n_{\mathrm{conv}}$, the convolution filter $W^{conv}$ is applied to $P_{i,j}^{\text{′}}$ to obtain the first feature maps $S_{i,j}^{1}\in \;R^{\left(T-n_{f}+1\right)\times \left(N_{1}-n_{d}+1\right)\times n_{\mathrm{conv}}}$. The area and process of convolution are defined as follows,

(11) $$P_{m,n}^{conv}=P_{i,j}^{\prime }\left(m:m+n_{f},\;n:n+n_{d}\right),$$

(12) $$\begin{array}{c} S_{i,j}^{1}\left(m,n,k\right)=\;g\left(W^{conv}\left(:,:,k\right)\times \;P_{m,n}^{conv}+b^{conv}\left(k\right)\right),\\ m\in \left[1,T-n_{f}+1\right],n\in \left[1,N_{1}-n_{d}+1\right],\;k\in \left[1,n_{\mathrm{conv}}\right], \end{array}$$

where $P_{m,n}^{conv}$ is the region covered by the sliding window when filter $W^{conv}$ slides to the *m*^th^ row and the *n*^th^ column of $P_{i,j}^{\text{′}}$. g(t)=ReLu(t)=max(0,t) is the activation function, and $b^{conv}\left(k\right)$ is the *k*^th^ bias vector. If convolution filter $W^{conv}$ is applied to the embedding matrix $P_{2,4}$ of node pairs $l_{2}$-$d_{4}$, the first feature map $S_{2,4}^{1}$ will be obtained.

Robust features can be extracted from feature map by applying max-pooling. In the pooling layer, the max-pooling operation was performed on $S_{i,j}^{1}$ to obtain the feature representation $Q_{i,j}^{1}\in \;R^{\left(T-n_{f}-n_{a}+2\right)\times \left(N_{1}-n_{d}-n_{b}+2\right)\times n_{\mathrm{conv}}}$,

(13) $$\begin{array}{c} Q_{i,j}^{1}\left(m,n,k\right)=\;\mathrm{MAX}\left(S_{i,j}^{1}\left(m:m+n_{a},\;n:n+n_{d},k\right)\right),\\ m\in \left[1,T-n_{f}-n_{a}+2\right],n\in \left[1,N_{1}-n_{d}-n_{b}+2\right],\;k\;\in \left[1,n_{\mathrm{conv}}\right], \end{array}$$

where $n_{a}$ and $n_{b}$ are the length and width of the pooling layer sliding window, respectively. $S_{i,j}^{1}\left(m:m+n_{a},\;n:n+n_{d},k\right)$ is the region covered by the sliding window when pooling window slides to the *m*^th^ row and the *n*^th^ column of $S_{i,j}^{1}$. Robust features are extracted from this region. If max-pooling was performed on the feature maps $S_{2,4}^{1}$ of node pair $l_{2}$-$d_{4}$, the feature representation $Q_{2,4}^{1}$ will be obtained. Next, we will continue to use node pairs $l_{2}$-$d_{4}$ as an example.

$Q_{2,4}^{1}$ was used as the input of the second convolution layer to obtain the feature representation $Q_{2,4}^{2}$ after the convolution and max-pooling operations. Convolution and max-pooling were also run on $Q_{2,4}^{2}$ in the third convolution layer and the pooling layer to obtain the feature representation $Q_{2,4}^{3}\in \;R^{n_{m}\times n_{g}\times n_{\mathrm{conv}}}$. $n_{m}$ and $n_{g}$ are respectively the length and width of the feature representation after three convolutions and pooling. $Q_{2,4}^{3}$ was flattened into the vector $q_{2,4}\;\in \;R^{n_{m}*n_{g}*n_{\mathrm{conv}}}$. Similarly, the fully connected and SoftMax layers served to project $q_{2,4}$ onto the C (C = 2)-associated probability distribution ***p^r^*** of class C (C = 2). The probability class 1 was taken as the predictive $score_{2,4}^{r}$ of the association between $l_{2}$ and $d_{4}$,

(14) $$score_{2,4}^{r}=\mathrm{softmax}\;\left(\;W^{r}q_{2,4}+b^{r}\right),$$

where $W^{r}\in \;R^{2\times \left(n_{m}*n_{g}*n_{\mathrm{conv}}\right)}$ is the parameter matrix of the fully connected layer and $b^{r}$ is the bias term. $score_{2,4}^{r}$ measures the probability of association between lncRNA $l_{2}$ and disease $d_{4}$. The higher its value is, the more likely the association is between them. The probability $score_{i,j}^{r}$ in which $l_{i}$ and $d_{j}$ may be correlated can be obtained by the same method.

### 2.3. Combination Strategy

The left and right sides of the model analyzed the relationship between lncRNA $l_{2}$ and disease $d_{4}$ from different perspectives. To combine their characteristics and improve model performance, a combination strategy was designed for the final prediction.

The cross-entropy loss between the association prediction distribution ***p^l^*** and the real distribution on the left side of the model is defined as follows,

(15) $$loss^{l}=\;-\;\sum_{i=1}^{T}\sum_{j=1}^{C}\;z_{j}\log\left(p_{j}^{l}\right),$$

where *T* is the number of training samples and *z* is the sample label. The cross-entropy loss on the right side of the model is defined as follows,

(16) $$loss^{r}=\;-\;\sum_{i=1}^{T}\sum_{j=1}^{C}\;z_{j}\log\left(p_{j}^{r}\right).$$

The final association prediction $score_{2,4}$ of $l_{2}$ and $d_{4}$ is the weighted sum of $score_{2,4}^{l}$ and $score_{2,4}^{r}$,

(17) $$score_{i,j}=\;\lambda \;\times \;score_{i,j}^{l}+\left(1\;-\;\lambda \right)\;\times score_{i,j}^{r}.$$

λ ∈(0 , 1) evaluates the contributions of the left and right sides of the model.

### 2.4. Reducing Overfitting

There are many parameters in our neural network. The higher the number of parameters, the easier it is to cause over-fitting. The recent technique, “dropout”, consists of setting the output of each hidden neuron to zero with a probability of 0.5. The neurons that are “dropped out” in this way do not participate in the forward pass and back-propagation [46]. Thus, every time an input is presented, the neural network samples a different architecture, but all these architectures share weights. This technique reduces intricate co-adaptation of neurons, because a neuron cannot depend on the existence of other neurons. Therefore, it is forced to learn robust and beneficial features in conjunction with different random subsets of other neurons. During the test, we multiplied the output of all the neurons by 0.5, which reasonably approximates the geometric mean of the predictive distributions produced exponentially by many dropout networks.

## 3. Results and Discussion

### 3.1. Performance Evaluation Metrics

We used fivefold cross-validation to evaluate and compare the performance of GCNLDA with other state-of-the-art prediction methods. If there is an association between lncRNA $l_{i}$ and disease $d_{j}$, then the node pair $l_{i}-d_{j}$ is regarded as a positive example. In contrast, the lack of association indicates that $l_{i}-d_{j}$ is a negative example. In the whole dataset, there were far fewer positive than negative examples. This discrepancy created a class imbalance affecting the model training. Therefore, we must randomly extract the same number of negative examples as the total number of positive samples from the dataset then randomly divide them into five equal subsets. All positive examples were also partitioned into five subsets of equal size. Four subsets each from the positive and negative examples were used to train the prediction model. All remaining samples were used for testing. Before each cross-validation, we removed the lncRNA-disease associations to be used for testing purposes then recalculated the similarity of the lncRNAs with the remaining associations.

We used the trained model to estimate the association prediction scores of the test samples then ranked them in descending order. When the association prediction score between lncRNA and disease was > **θ** (a threshold), this example was deemed positive. Otherwise, it was scored as a negative example. We used TP and TN to represent the numbers of correctly identified positive and negative example, respectively. FN and FP represented the numbers of misidentified positive and negative examples, respectively. The TPR (true positive rate), FPR (false positive rate), Precision (precision), and Recall (recall rate) were calculated as follows,

(18) $$TPR=\;\frac{TP}{TP+FN},\;FPR=\;\frac{FP}{TN+FP},$$

(19) $$Precision=\;\frac{TP}{TP+FP},\;Recall=\;\frac{TP}{TP+FN}.$$

The TPRs, FPRs, Precisions, and Recalls were calculated by changing **θ**. The TPRs and FPRs were used to plot the receiver operating characteristic (ROC) curve. The area under the ROC curve (AUC) was used to measure the global performance of the prediction method. To improve the assessment of the model performance in the event of class imbalance, we plotted the precision-recall (PR) curve based on the calculated precisions and recalls. The area under the PR curve (AUPR) also quantified the overall performance of the prediction method. GCNLDA’s AUCs and AUPRs during each cross-validation are listed in Supplementary Table S1.

The preceding equation shows that recall is the ratio of correctly identified positive examples to all positive examples. The number of positive examples appearing as top k lncRNA candidates of the disease increases with the corresponding recall. Researchers usually select the top-ranked candidates from the prediction results for experimental verification. Thus, it is reasonable to use high Recall values. Therefore, we also calculated the recall values of the top 30, 60, 90…210, 240 candidates for ten diseases.

### 3.2. Comparison with Other Methods

GCNLDA’s hyperparameters, λ, *n*, $n_{\mathrm{conv}1}$, $n_{\mathrm{conv}2}$, $n_{\mathrm{conv}3}$, $n_{f\;}$, and $n_{d}$ were tuned. The values of λ and *n* were selected from {0.1, 0.2, 0.3, 0.4, 0.5, 0.6, 0.7, 0.8, 0.9} and {50, 100, 200, 300, 400, 500}, respectively. The values of $n_{\mathrm{conv}1}$, $n_{\mathrm{conv}2}$, $n_{\mathrm{conv}3}$ were selected from {5, 10, 20, 30, 40, 50, 60, 70}. The $n_{f\;}$, $n_{d}$ values were selected from {1, 2, 3, 5, 7, 9, 11, 12, 14, 16, 18, 20}. GCNLDA’s yielded the best performance when λ =0.8, *n* = 100, $n_{\mathrm{conv}1}=20$, $n_{\mathrm{conv}2}=30$, $n_{\mathrm{conv}3}=40$, $n_{f\;}=3$ and $n_{d}=11$. The optimal set parameters were obtained using a grid search.

In order to evaluate the ability of our model to predict lncRNA-disease associations, we compared it with other state-of-the-art prediction methods including Ping’s method [25], LDAP [32], MFLDA [33], and SIMCLDA [34]. We adjusted the parameters of GCNLDA based on the cross-validation to optimize its prediction performance. On the left side of the model, network node representations with n = 100 were obtained from the graph convolution encoding operation. The learning rate of the autoencoder was set to 0.001. On the right side of the model, $n_{\mathrm{conv}1}$ = 20 filters, $n_{\mathrm{conv}2}$ = 30 filters, and $n_{\mathrm{conv}3}$ = 40 filters of length $n_{f\;}$ = 3 and width $n_{d}$ = 11 were used in three convolution layers. The learning rate was set to 0.0005. The parameters were updated by the Adam optimization algorithm throughout the training process. ReLu was the activation function for all fully connected layers. The optimal parameters of other methods are obtained through grid search. For SIMCLDA, $\alpha_{l}$ = 0.8, $\alpha_{d}$ = 0.6, and λ = 1; for Ping’s method, α = 0.6; for MFLDA, α = 10^5^; for LDAP, gap open = 10, and gap extend = 0.5.

**Figure 4:**
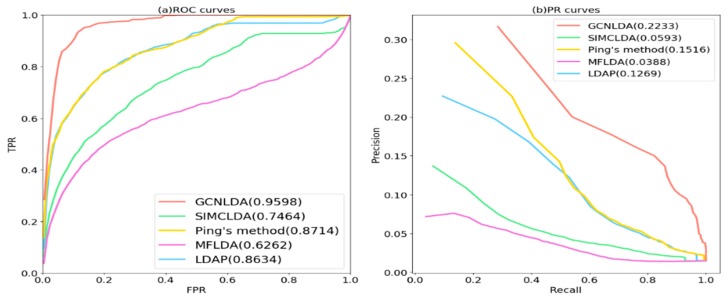
Receiver operating characteristic (ROC) and precision-recall (PR) curves of GCNLDA and other methods for all diseases. (**a**) ROC curves of all the methods; (**b**) PR curves of all the methods.

As shown in Figure 4a and Table 1, GCNLDA had the best performance for 405 diseases. The AUC of the ROC curve was 0.959. The performance of GCNLDA was superior to those of SIMCLDA, Ping’s method, MFLDA, and LDAP by 21.34%, 8.84%, 33.36%, and 9.64%, respectively. We listed the AUC of all five methods based on 10 well-characterized diseases. Each of these has > 15 known lncRNAs associated with them. GCNLDA presented with the best performance on these 10 diseases (Table 1). Ping’s method and LDAP fused the similarity of lncRNA and disease which improved the accuracy of their similarity calculations and achieved good performance. Ping’s method also exploited the topology information of the bipartite networks so its performance was slightly superior to that of LDAP. In contrast, SIMCLDA only fused multiple similarities of lncRNA. Consequently, its performance was inferior to those of the aforementioned methods. MFLDA integrates multiple data sources but ignores the similarity of lncRNAs and diseases. As a result, its performance is inferior to those of the other methods. The aforementioned methods focus mainly on lncRNA, disease similarity, and integration of multiple data sources. They make negligible use of network topology information. The advantages of GCNLDA over the other methods include deep learning to extract the local representation of lncRNA-disease node pairs and graph convolution to learn their network representation.

As shown in Figure 4b and Table 2, GCNLDA had the best performance for 405 diseases (AUPR = 0.2233). It was 16.4% better than SIMCLDA, 7.17% better than Ping’s method, 18.45% better than MFLDA, and 9.64% better than LDAP. GCNLDA achieved the best performance for nine of the ten well-characterized diseases.

To verify whether the performance of our method was significantly better than those of the other methods, we conducted paired Wilcoxon tests on GCNLDA and the others. In all cases, *p* < 0.05 (Table 3). Relative to the other methods, then, the performance of GCNLDA in the AUPRs and AUCs was significantly better.

**Figure 5:**
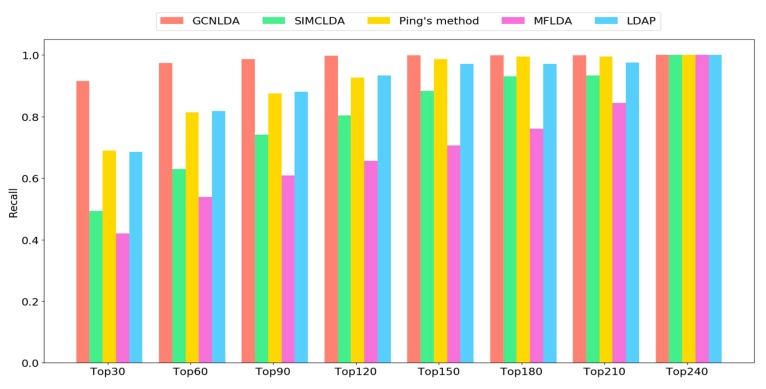
Average recalls across all tested diseases under different top *k* cutoffs.

As shown in Figure 5, the recall rate on the top *k* ranked lncRNAs increases with the number of correctly identified known lncRNA-disease associations. GCNLDA consistently outperformed other methods at different *k* values. The average recall rates of the top 30, 60, 90, and 120 lncRNA candidates for GCNLDA were 91.5%, 97.3%, 98.5%, and 99.7%, respectively. For Ping’s method, they were 68.9%, 81.3%, 87.5%, and 92.7%, respectively. For LDAP, they were 68.5%, 81.3%, 88%, and 93.3%, respectively. For SIMCLDA, they were 49.3%, 63%, 74.1%, and 80.3%, respectively. For MFLDA, they were 42%, 53.9%, 61%, and 65.5%, respectively.

### 3.3. Case Studies on Stomach Cancer, Osteosarcoma, and Lung Cancer

To test the ability of GCNLDA to predict potential lncRNA-disease associations, we conducted a case analysis on stomach cancer, osteosarcoma, and lung cancer. We analyzed in detail the top 15 candidates for related diseases (Table 4). The top 15 candidates for all the 405 diseases were obtained through GCNLDA and are listed in Supplementary Table S2. All known lncRNA-disease associations were treated as training samples and all lncRNA-disease pairs with unknown associations were used as test samples.

Lnc2Cancer is an experimentally corroborated database consisting of 4986 lncRNA-disease associations. It includes 1614 human lncRNAs and 165 human cancers. The database LncRNADisease contains lncRNA-disease associations verified by experimentation and predicted by state-of-the-art methods. Twelve of the 15 lncRNA candidates related to stomach cancer were included in the Lnc2Cancer database and 10 of them were included among the experimentally verified data in LncRNADisease. The databases confirmed whether the lncRNAs were associated with stomach cancer. If the disease-related lncRNA candidate was labelled as “Literature”, then it was supported in published studies. As shown in Table 4, candidate MIR17HG (alias mir-17-92) was labelled as “Literature” and proved to be dysregulated in stomach cancer [47].

Among the top 15 lncRNA candidates of osteosarcoma listed in Table 4, ten were included in the Lnc2Cancer database whilst two were queried in LncRNADisease with experimental support. They were confirmed to have definite associations with osteosarcoma. Recently published studies showed that AFAP1-AS1 enhances cell proliferation and invasion in osteosarcoma by regulating miR-4695-5p/TCF4-β-catenin signaling [48]. Nine of the top 15 lncRNA candidates of lung cancer were in Lnc2Cancer and eight appeared in LncRNADisease. Recent reports confirmed that lncRNA MIR155HG promotes lung cancer cell proliferation, migration, and invasion [49].

The remaining eight lncRNA candidates labelled “LncRNADisease*” were included in the predicted lncRNA-disease associations in the LncRNADisease database. These predictions reveal that GCNLDA effectively discovers potential lncRNA-disease associations.

## 4. Conclusions

GCNLDA predicts potential lncRNA-disease associations and it is based on graph convolutional network and convolutional neural networks. Attention mechanism was constructed at the node feature level to distinguish the various contributions of the node features. The graph convolution autoencoder with an attention mechanism deeply integrates the topological information of lncRNA-disease-miRNA heterogeneous networks. The convolutional neural network module captures various connection relationships related to lncRNA-disease on the node pair embedding. The network and local representations of lncRNA-disease node pairs were learned by the new framework based on graph convolutional network and convolutional neural networks. Cross-validation confirmed that GCNLDA is superior to other state-of-the-art methods in terms of both AUC and AUPR. Case studies on three diseases substantiated the ability of GCNLDA to predict potential disease-associated lncRNAs. GCNLDA may serve as an effective tool to screen reliable candidates for lncRNA-disease association validation with-lab experiment.

## Figures and Tables

**Table 1:** Area under the ROC curves (AUCs) of GCNLDA and other methods for all the diseases and 10 well-characterized diseases.

Disease Name	AUC				
	GCNLDA	SIMCLDA	Ping’s Method	MFLDA	LDAP
Average AUC on 405 diseases	**0.959**	0.746	0.871	0.626	0.863
respiratory system cancer	**0.948**	0.789	0.911	0.719	0.891
organ system cancer	**0.992**	0.82	0.95	0.729	0.884
intestinal cancer	**0.966**	0.811	0.909	0.559	0.905
prostate cancer	**0.944**	0.873	0.826	0.553	0.71
lung cancer	**0.961**	0.79	0.911	0.676	0.883
breast cancer	**0.963**	0.742	0.871	0.517	0.83
reproductive organ cancer	**0.962**	0.707	0.818	0.74	0.742
gastrointestinal system cancer	**0.977**	0.784	0.896	0.582	0.867
liver cancer	**0.978**	0.799	0.91	0.634	0.898
hepatocellular carcinoma	**0.983**	0.765	0.903	0.688	0.902

The bold values indicate the higher AUCs.

**Table 2:** AUPRs of GCNLDA and other methods for all the diseases and 10 well-characterized diseases.

Disease Name	AUPR				
	GCNLDA	SIMCLDA	Ping’s Method	MFLDA	LDAP
Average AUC on 405 diseases	**0.223**	0.166	0.219	0.095	0.066
respiratory system cancer	**0.465**	0.149	0.414	0.072	0.303
organ system cancer	**0.950**	0.411	0.765	0.338	0.628
intestinal cancer	**0.697**	0.141	0.252	0.042	0.246
prostate cancer	**0.594**	0.176	0.333	0.095	0.297
lung cancer	**0.600**	0.138	0.334	0.008	0.094
breast cancer	0.623	0.445	**0.803**	0.476	0.629
reproductive organ cancer	**0.625**	0.047	0.403	0.031	0.396
gastrointestinal system cancer	**0.812**	0.130	0.271	0.104	0.238
liver cancer	**0.671**	0.201	0.526	0.086	0.498
hepatocellular carcinoma	**0.787**	0.096	0.239	0.082	0.303

The bold values indicate the higher AUPRs.

**Table 3:** A pairwise comparison with a paired Wilcoxon-test on the prediction results.

*p*-Value	SIMCLDA	Ping’s Method	MFLDA	LDAP
*p*-value of ROC curve	1.131026 × 10^−106^	1.494908 × 10^−44^	4.534043 × 10^−124^	4.291344 × 10^−50^
*p*-value of PR curve	1.342560 × 10^−89^	2.204929 × 10^−29^	1.567472 × 10^−112^	2.844473 × 10^−48^

**Table 4:** The top 15 candidate lncrnas for stomach cancer, osteosarcoma and lung cancer.

Disease Name	Rank	lncRNA	Evidence	Rank	lncRNA	Evidence
Stomach cancer	1	MALAT1	Lnc2Cancer, LncRNADisease	9	HULC	Lnc2Cancer, LncRNADisease
	2	NEAT1	Lnc2Cancer, LncRNADisease	10	CCAT2	Lnc2Cancer, LncRNADisease
	3	MIR17HG	Literature [47]	11	KCNQ1OT1	Lnc2Cancer
	4	HOTTIP	Lnc2Cancer, LncRNADisease	12	BCYRN1	LncRNADisease*
	5	TUG1	Lnc2Cancer, LncRNADisease	13	CASC2	Lnc2Cancer, LncRNADisease
	6	HNF1A-AS1	Lnc2Cancer, LncRNADisease	14	PANDAR	Lnc2Cancer, LncRNADisease
	7	XIST	Lnc2Cancer, LncRNADisease	15	PCAT1	LncRNADisease*
	8	AFAP1-AS1	Lnc2Cancer			
Osteosarcoma	1	H19	Lnc2Cancer, LncRNADisease	9	LINC00675	LncRNADisease*
	2	GAS5	Lnc2Cancer	10	BCYRN1	LncRNADisease*
	3	PVT1	Lnc2Cancer	11	CCAT2	Lnc2Cancer
	4	NEAT1	Lnc2Cancer	12	CASC2	Lnc2Cancer
	5	EWSAT1	Lnc2Cancer	13	CCAT1	Lnc2Cancer
	6	AFAP1-AS1	Literature [48]	14	TP73-AS1	Lnc2Cancer
	7	CDKN2B-AS1	LncRNADisease	15	PCA3	LncRNADisease*
	8	SPRY4-IT1	Lnc2Cancer			
Lung cancer	1	KCNQ1OT1	Lnc2Cancer	9	IGF2-AS	Lnc2Cancer
	2	HOTTIP	Lnc2Cancer, LncRNADisease	10	PCAT1	LncRNADisease
	3	SPRY4-IT1	Lnc2Cancer, LncRNADisease	11	CASC2	Lnc2Cancer, LncRNADisease
	4	TP73-AS1	Lnc2Cancer	12	ESRG	LncRNADisease*
	5	MIAT	Lnc2Cancer	13	PCA3	LncRNADisease*
	6	MIR155HG	Literature [49]	14	SNHG12	Lnc2Cancer
	7	LINC00675	LncRNADisease*	15	TUSC7	Lnc2Cancer
	8	SOX2-OT	LncRNADisease			

“Lnc2Cancer” means the lncRNA candidate was included in the Lnc2Cancer database. “LncRNADisease” means the candidate was included among the experimentally verified data in LncRNADisease. “LncRNADisease*” means the candidate was included among the predicted data in LncRNADisease. “Literature” means the candidate was supported in published studies.

## Supplementary Materials

The following are available online at https://www.mdpi.com/2073-4409/8/9/1012/s1, Table S1: AUC and AUPR of GCNLDA in each cross-validation. Table S2: The top 15 potential lncRNA candidates for 405 diseases.

## References

[1] Taft R.J., Pang K.C., Mercer T.R., Dinger M.E., Mattick J.S. (2010). Non-coding RNAs: Regulators of disease. J. Pathol..

[2] Chen X., Yan C.C., Zhang X., You Z.H. (2017). Long non-coding RNAs and complex diseases: From experimental results to computational models. Briefings Bioinform..

[3] Harrow J., Frankish A., Gonzalez J.M., Tapanari E., Diekhans M., Kokocinski F., Aken B.L., Barrell D., Zadissa A., Searle S. (2012). GENCODE: The reference human genome annotation for the ENCODE project. Genome Res..

[4] Marcia G., Danielle M., Buddy S.H., Dorssers L.C.J., Ton V.A. (2011). Characterization of BCAR4, a novel oncogene causing endocrine resistance in human breast cancer cells. J. Cell. Physiol..

[5] Hrdlickova B., Almeida R.C.D., Borek Z., Withoff S. (2014). Genetic variation in the non-coding genome: Involvement of micro-RNAs and long non-coding RNAs in disease. BBA Mol. Basis Dis..

[6] Ada C., Kei K., Ryousuke O., Osamu Y., Keishi M., Eiichiro Y., Tatsuo K., Hiroshi K., Hiroko Y., Yasushi T. (2012). Genetic variants at the 9p21 locus contribute to atherosclerosis through modulation of ANRIL and CDKN2A/B. Atherosclerosis.

[7] Johnson R. (2012). Long non-coding RNAs in Huntington’s disease neurodegeneration. Neurobiol. Dis..

[8] Mamoshina P., Vieira A., Putin E., Zhavoronkov A. (2016). Applications of deep learning in biomedicine. Mol. Pharm..

[9] Zhang T., Wang M., Xi J., Ao L. (2018). LPGNMF: Predicting long non-coding RNA and protein interaction using graph regularized nonnegative matrix factorization. IEEE/ACM Trans. Comput. Biol. Bioinform..

[10] Piro R.M., Marsico A., Lai X., Gupta S.K., Vera J. (2019). network-based methods and other approaches for predicting lncRNA functions and disease associations. Computational Biology of Non-Coding RNA: Methods and Protocols.

[11] Fu L., Peng Q. (2017). A deep ensemble model to predict miRNA-disease association. Sci. Rep..

[12] Bressin A., Schulte-Sasse R., Figini D., Urdaneta E.C., Beckmann B.M., Marsico A. (2019). TriPepSVM: De novo prediction of RNA-binding proteins based on short amino acid motifs. Nucleic Acids Res..

[13] Heller D., Krestel R., Ohler U., Vingron M., Marsico A. (2017). ssHMM: Extracting intuitive sequence-structure motifs from high-throughput RNA-binding protein data. Nucleic Acids Res..

[14] Budach S., Marsico A. (2018). pysster: Classification of biological sequences by learning sequence and structure motifs with convolutional neural networks. Bioinformatics.

[15] Krakau S., Richard H., Marsico A. (2017). PureCLIP: Capturing target-specific protein–RNA interaction footprints from single-nucleotide CLIP-seq data. Genome Biol..

[16] Chen G., Wang Z., Wang D., Qiu C., Liu M., Chen X., Zhang Q., Yan G., Cui Q.J.N.A.R. (2012). LncRNADisease: A database for long-non-coding RNA-associated diseases. Nucleic Acids Res..

[17] Li J.W., Gao C., Wang Y.C., Ma W., Tu J., Wang J.P., Chen Z.Z., Kong W., Cui Q.H. (2014). A bioinformatics method for predicting long noncoding RNAs associated with vascular disease. Sci. China Life Sci..

[18] Ming-Xi L., Xing C., Geng C., Qing-Hua C., Gui-Ying Y. (2014). A computational framework to infer human disease-associated long noncoding RNAs. PLoS ONE.

[19] Biswas A.K., Zhang B., Wu X., Gao J.X. (2015). A multi-label classification framework to predict disease associations of long non-coding RNAs (lncRNAs). Proceedings of the Third International Conference on Communications, Signal Processing, and Systems.

[20] Chen X., Yan G.-Y. (2013). Novel human lncRNA-disease association inference based on lncRNA expression profiles. Bioinformatics.

[21] Chen X., Yan C.C., Luo C., Ji W., Zhang Y., Dai Q. (2015). Constructing lncRNA functional similarity network based on lncRNA-disease associations and disease semantic similarity. Sci. Rep..

[22] Xing C., Yuan H., Wang X.S., You Z.H., Chan K.C.C. (2016). FMLNCSIM: fuzzy measure-based lncRNA functional similarity calculation model. Oncotarget.

[23] Huang Y.A., Chen X., You Z.H., Huang D.S., Chan K.C.C. (2016). ILNCSIM: improved lncRNA functional similarity calculation model. Oncotarget.

[24] Xiaofei Y., Lin G., Xingli G., Xinghua S., Hao W., Fei S., Bingbo W. (2014). A network based method for analysis of lncRNA-disease associations and prediction of lncRNAs implicated in diseases. PLoS ONE.

[25] Ping P., Wang L., Kuang L., Ye S., Mfb I., Pei T. (2018). A novel method for lncRNA-disease association prediction based on an lncRNA-disease association network. IEEE/ACM Trans. Comput. Biol. Bioinform..

[26] Jie S., Hongbo S., Zhenzhen W., Changjian Z., Lin L., Letian W., Weiwei H., Dapeng H., Shulin L., Meng Z. (2014). Inferring novel lncRNA-disease associations based on a random walk model of a lncRNA functional similarity network. Mol. Biosyst..

[27] Chen X., You Z.H., Yan G.Y., Gong D.W. (2016). IRWRLDA: Improved random walk with restart for lncRNA-disease association prediction. Oncotarget.

[28] Gu C., Liao B., Li X., Cai L., Li Z., Li K., Yang J. (2017). Global network random walk for predicting potential human lncRNA-disease associations. Sci. Rep..

[29] Yu G., Fu G., Chang L., Ren Y., Wang J. (2017). BRWLDA: Bi-random walks for predicting lncRNA-disease associations. Oncotarget.

[30] Yao Q., Wu L., Li J., Yang L.G., Sun Y., Li Z., He S., Feng F., Li H., Li Y. (2017). Global prioritizing disease candidate lncRNAs via a multi-level composite network. Sci. Rep..

[31] Pooya Z., Ben J., Raf V., Yves M. (2014). Protein fold recognition using geometric kernel data fusion. Bioinformatics.

[32] Lan W., Li M., Zhao K., Liu J., Wu F.X., Pan Y., Wang J. (2017). LDAP: A web server for lncRNA-disease association prediction. Bioinformatics.

[33] Fu G., Wang J., Domeniconi C., Yu G. (2017). Matrix factorization based data fusion for the prediction of lncRNA-disease associations. Bioinformatics.

[34] Lu C., Yang M., Luo F., Wu F.X., Li M., Pan Y., Li Y., Wang J. (2018). Prediction of lncRNA-disease associations based on inductive matrix completion. Bioinformatics.

[35] Ning S., Zhang J., Wang P., Zhi H., Wang J., Liu Y., Gao Y., Guo M., Yue M., Wang L. (2016). Lnc2Cancer: A manually curated database of experimentally supported lncRNAs associated with various human cancers. Nucleic Acids Res..

[36] Lu Z., Cohen K.B., Hunter L. (2007). GeneRIF quality assurance as summary revision. Proceedings of the Pacific Symposium on Biocomputing.

[37] Li J., Liu S., Zhou H., Qu L., Yang J. (2014). starBase v2.0: Decoding miRNA-ceRNA, miRNA-ncRNA and protein–RNA interaction networks from large-scale CLIP-Seq data. Nucleic Acids Res..

[38] Li Y., Qiu C., Tu J., Geng B., Yang J., Jiang T., Cui Q. (2014). HMDD v2.0: A database for experimentally supported human microRNA and disease associations. Nucleic Acids Res..

[39] Cheng L., Hu Y., Sun J., Zhou M., Jiang Q. (2018). DincRNA: A comprehensive web-based bioinformatics toolkit for exploring disease associations and ncRNA function. Bioinformatics.

[40] Wang D., Wang J., Lu M., Song F., Cui Q. (2010). Inferring the human microRNA functional similarity and functional network based on microRNA-associated diseases. Bioinformatics.

[41] Kipf T.N., Welling M. Semi-supervised classification with graph convolutional networks. Proceedings of the ICLR 2017.

[42] Zitnik M., Agrawal M., Leskovec J. (2018). Modeling polypharmacy side effects with graph convolutional networks. Intell. Syst. Mol. Biol..

[43] Pan S., Hu R., Fung S., Long G., Jiang J., Zhang C. Learning Graph Embedding with Adversarial Training Methods. https://arxiv.org/abs/1901.01250.

[44] Den Berg R.V., Kipf T.N., Welling M. Graph convolutional matrix completion. Proceedings of the KDD’18 Deep Learning Day.

[45] Torng W., Altman R.B. (2018). Graph convolutional neural networks for predicting drug-target interactions. bioRxiv.

[46] Hinton G.E., Srivastava N., Krizhevsky A., Sutskever I., Salakhutdinov R.R. Improving Neural Networks by Preventing Co-Adaptation of Feature Detectors. https://arxiv.org/abs/1207.0580v1.

[47] Bahari F., Emadibaygi M., Nikpour P. (2015). miR-17-92 host gene, uderexpressed in gastric cancer and its expression was negatively correlated with the metastasis. Indian J. Cancer.

[48] Li R., Liu S., Li Y., Tang Q., Xie Y., Zhai R. (2018). Long noncoding RNA AFAP1-AS1 enhances cell proliferation and invasion in osteosarcoma through regulating miR-4695-5p/TCF4-β-catenin signaling. Mol. Med. Rep..

[49] Sun B., Yang N. (2017). Long non-coding RNA MIR155HG promotes proliferation, migration and invasion of A549 human lung cancer cells. J. Chongqing Med. Univ..

