# Supplementary material for: Graph Convolutional Network and Convolutional Neural Network Based Method for Predicting lncRNA-Disease Associations
Source: Cells. 2019 Aug 30;8(9):1012. doi: 10.3390/cells8091012 (PMC6769579; doi:10.3390/cells8091012)
Supplement: Supplementary file 1 [file cells-08-01012-s001.zip › Table S1.pdf]

Table S1. AUC and AUPR of GCNLDA in each cross-validation.

NO.	AUC	AUPR
1	0.961	0.225
2	0.957	0.222
3	0.959	0.223
4	0.962	0.226
5	0.956	0.219
